# Estrogen related receptor alpha in castration-resistant prostate cancer cells promotes tumor progression in bone

**DOI:** 10.18632/oncotarget.12787

**Published:** 2016-10-20

**Authors:** Anais Fradet, Mathilde Bouchet, Carine Delliaux, Manon Gervais, Casina Kan, Claire Benetollo, Francesco Pantano, Geoffrey Vargas, Lamia Bouazza, Martine Croset, Yohann Bala, Xavier Leroy, Thomas J Rosol, Jennifer Rieusset, Akeila Bellahcène, Vincent Castronovo, Jane E Aubin, Philippe Clézardin, Martine Duterque-Coquillaud, Edith Bonnelye

**Affiliations:** ^1^ InsermUMR1033, F-69372 Lyon, France; ^2^ Université-Lyon1, F-69008 Lyon, France; ^3^ CNRS-UMR8161, F-59021 Lille, France; ^4^ Université-Lille, F-59000 Lille, France; ^5^ InsermU1028-CNRS-UMR5292, Lyon, France; ^6^ University-Campus-Bio-Medico, 00128 Rome, Italy; ^7^ Centre Hospitalier Lille, F-59037 Lille, France; ^8^ College of Veterinary Medicine, Columbus, OH 43210, USA; ^9^ InsermUMR-U1060, F-69921 Oullins, France; ^10^ University Liege, B-4000 Liege, Belgium; ^11^ University of Toronto, Toronto, ON M5S 1A8, Canada

**Keywords:** ERRα, bone, prostate cancer, microenvironment

## Abstract

Bone metastases are one of the main complications of prostate cancer and they are incurable. We investigated whether and how estrogen receptor-related receptor alpha (*ERRα*) is involved in bone tumor progression associated with advanced prostate cancer. By meta-analysis, we first found that *ERRα* expression is correlated with castration-resistant prostate cancer (CRPC), the hallmark of progressive disease. We then analyzed tumor cell progression and the associated signaling pathways in gain-of-function/loss-of-function CRPC models *in vivo* and *in vitro*. Increased levels of ERRα in tumor cells led to rapid tumor progression, with both bone destruction and formation, and direct impacts on osteoclasts and osteoblasts. *VEGF-A*, *WNT5A* and *TGFβ1* were upregulated by *ERRα* in tumor cells and all of these factors also significantly and positively correlated with *ERRα* expression in CRPC patient specimens. Finally, high levels of ERRα in tumor cells stimulated the pro-metastatic factor *periostin* expression in the stroma, suggesting that *ERRα* regulates the tumor stromal cell microenvironment to enhance tumor progression. Taken together, our data demonstrate that *ERRα* is a regulator of CRPC cell progression in bone. Therefore, inhibiting *ERRα* may constitute a new therapeutic strategy for prostate cancer skeletal-related events.

## INTRODUCTION

Bone metastases are a frequent complication of cancer occurring in up to 80% of patients with advanced prostate cancer (PCa) and castration-resistance (CRPC “castration-resistant prostate cancer”) with associated poor five-year survival rate [1, 2]. They are not curable and result in impaired mobility and pathological fractures [3]. To grow in bone, tumor cells alter bone formation and resorption by secreting proteins that directly affect osteoblasts (bone-forming cells) and osteoclasts (bone-resorbing cells) resulting in the development of mixed lesions [1, 4]. These signaling proteins may include RANKL (receptor activator of the NF-kB ligand) which stimulates osteoclast differentiation [1, 5] and osteoprotegerin (OPG) which acts as a decoy receptor for RANKL receptor and inhibits osteoclastogenesis [5]. Therefore, the balance between RANKL and OPG is critical in controlling osteoclast activity and osteolysis in bone metastases. PCa cells also express factors such as TGFβ (transforming growth factor beta), WNT family members such as Wnt5a and the pro-angiogenic factor VEGFA that promote an aggressive tumor phenotype and bone metastases by directly affecting osteoclast and osteoblast formation [6, 7]. The induction of stromal niche signals by tumor cells, for example expression of extracellular matrix proteins such as PERIOSTIN (POSTN) in the tumor microenvironment, also contributes to the expansion of the metastatic niches [8–11].

Nuclear receptors are transcription factors that comprise ligand-dependent molecules, such as estrogen receptors (ERs), and a large number of so-called orphan receptors for which no ligand has yet been determined [12]. Estrogen receptor-related receptor alpha (ERRα)(NR3B1) shares structural similarities with ERα and ERβ (NR3A1/NR3A2) [13] but does not bind estrogen [14]. Since very recently, ERRα was considering as the oldest orphan receptor but Wei *et al.* just described the cholesterol as a potential ERRα agonist [15]. Synthetic molecules like the inverse agonist XCT-790 were also designed to block ERRα activity by preventing its interaction with the co-activators peroxisome proliferator-activated receptor gamma coactivator (PGC1) [16].

ERRα is expressed in a range of cancer cell types and ERRα-positive tumors (breast and prostate) are associated with more invasive disease and higher risk of recurrence [17, 18]. Indeed in prostate cancer, ERRα is significantly higher in cancerous lesions compared to benign foci and high level of ERRα correlates with Gleason score and poor survival [18]. Moreover, in androgen receptor (AR)-positive models, ERRα has been implicated in AR signaling pathways and shown to increase HIF-1 signaling and to promote hypoxic growth adaptation of prostate cancer cells [19, 20]. ERRα is also expressed in bone where it regulates differentiation and activity of osteoblasts and osteoclasts, both of which are implicated into the mixed osteolytic and osteoblastic lesions observed in advanced prostate cancer patients [15] [21]. Based on our previous data in bone metastases from breast cancer [22], and on the fact that bone metastases are the hallmark of progressive disease and CRPC, mainly characterized by AR alterations [23], we investigated whether and how ERRα is involved in bone progression of CRPC (AR-negative) models.

## RESULTS

### ERRα is more highly expressed in CRPC patients and their associated bone metastases than normal prostate and non-metastasizing PCa

**Figure 1:**
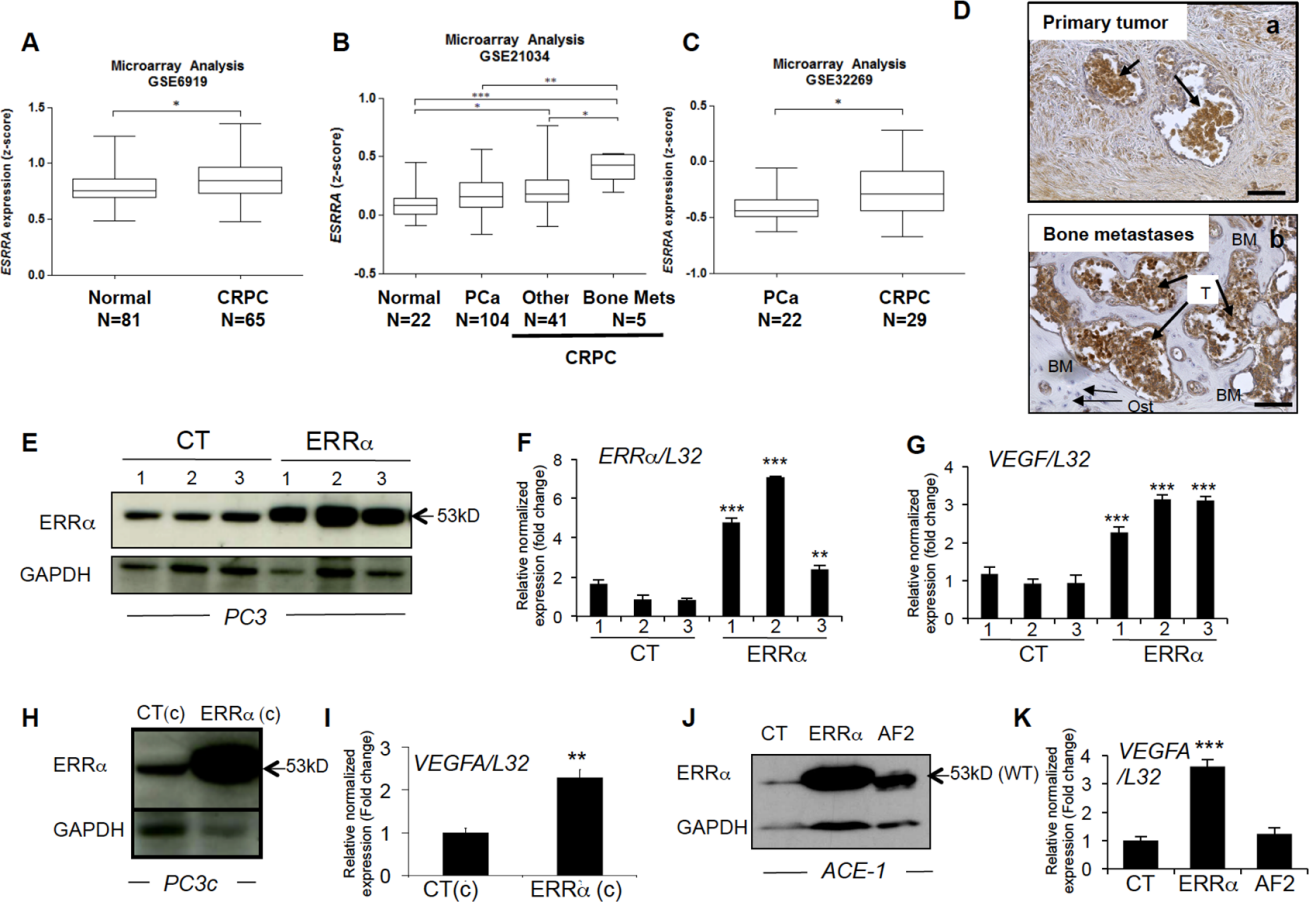
ERRα expression and CRPC from PCa patients (**A**) Meta-analysis using public datasets showed that *ERRα* mRNA expression is higher in CRPC patients in GSE6919 (Student's *t*-test *P* = 0.0172). (**B**) *ERRα* was also found to be higher in CRPC compared to androgen-sensitive PCa, as well as in primary tumors from CRPC patients that developed metastases to bone compared to other sites or normal prostate tissues in GSE21034 (One way ANOVA, bonferri post-hoc test : *P* < 0,05, normal (*n* = 22) versus CRPC (*n* = 41); *P* < 0.0005, normal (*n* = 22) versus CRPC bone mets (*n* = 5); *P* < 0.005, PCa (*n* = 104) versus CRPC bone mets (*n* = 5)) and (**C**) PCa versus CRPC (that all had developed bone metastases) in GSE32269 (Student's *t*-test *P* = 0.0178): *P < 0.05, **P < 0.005, ***P < 0.0005. (**D**) Visualization of *ERRα* protein expression by IHC on sections of prostate primary tumor (a) and the associated bone metastatic lesions (b) from the same patient. (**E**) Assessment of *ERRα* expression by Western blotting and (**F**) real-time RT-PCR on triplicate samples and normalized against the ribosomal protein gene *L32* (ANOVA, Student's *t*-tests *P* < 0.0001) in PC3 control (CT-1-3) and PC3-ERRα (*ERRα*-1–3) overexpressing *ERRα* clones. (**G**) Increased expression of *VEGF-A* mRNA in PC3-ERRα (ANOVA, Student's *t*-tests *P* < 0.0001). (**H**) Increase of ERRα protein expression in PC3c-ERRα (ERRα(c)) overexpressing ERRα shown by Western blot and (**I**) by real-time RT-PCR for *VEGF-A* expression (Student's *t*-tests *P* = 0.001). (**J**) Assessment of *ERRα* expression by Western blotting in an ACE-1 empty-vector CT clone, an ACE-ERRα and a clone overexpressing the dominant negative ERRα with AF2 domain deletion (AF2). (**K**) *VEGF-A* mRNA expression was also increased in ACE-ERRα cells (Student's *t*-tests *P* = 0.0001). Bar = 200 μm, T: Tumor; Ost: osteocytes; BM: Bone Matrix

To determine whether ERRα is involved in PCa bone lesions, we first assessed *ERR*α mRNA expression (*ESRRA*) levels during disease progression by performing a meta-analysis of data from the gene expression omnibus (GEO; GSE69129, GSE21034 and GSE32269) (Figure 1A–1C, Supplementary Table S1) [24, 25, 26]. We found that *ERR*α expression was significantly higher in CRPC compared to normal prostate (*P* = 0.0172)(Figure 1A) and (*P* = < 0.05, *n* = 22 (normal) *vs n* = 41 (CRPC)) (Figure 1B). Higher *ERR*α expression was also observed in primary tumors from CRPC patients who had developed bone metastases compared to androgen-sensitive PCa patients (*P* < 0.005, (PCa) *vs* (CRPC bone Mets))(Figure 1B) and (*P* = 0.0178, (PCa) *vs* (CRPC who all developed bone metastases)) (Figure 1C). In the dataset GSE21034, we also found that *ERR*α mRNA was significantly higher in primary cancerous prostate lesions from CRPC who developed bone metastatic lesions (*n* = 5) compared to patients with had developed other types of metastases (brain, lung, bladder, colon or lymph nodes) (*n* = 41) (*P* < 0.05; Figure 1B) suggesting that *ERR*α is associated with advanced prostate cancer and bone metastases. Immunohistochemistry also revealed that *ERRα* protein expression in human PCa cells was maintained in the associated bone metastases (Figure 1D), suggesting that ERRα is an overall poor prognostic factor for bone metastases from CRPC.

### ERRα in PCa cells promotes tumor cells progression *in vivo* in bone microenvironment

**Figure 2:**
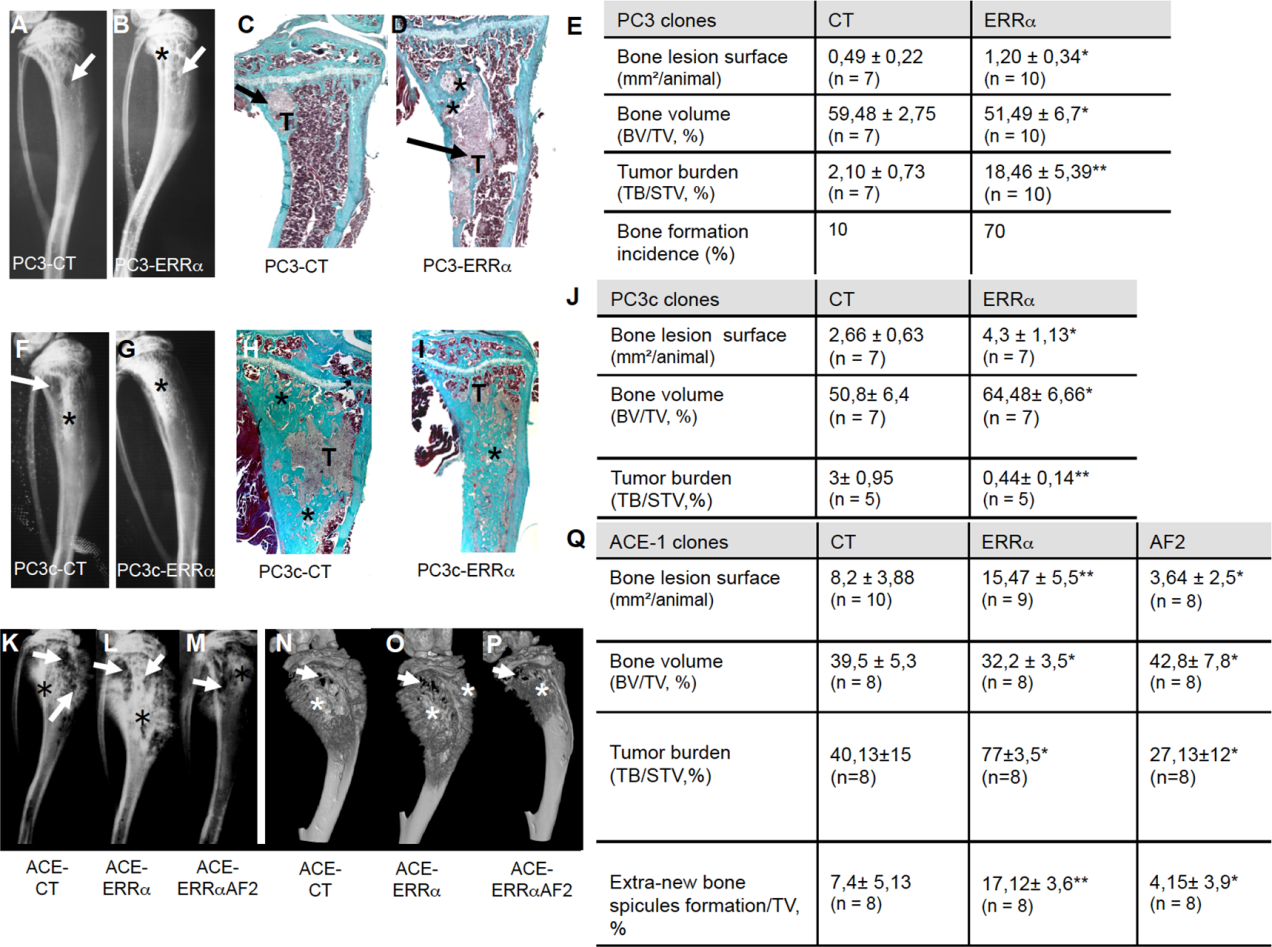
Over-expression of *ERRα* in prostate cancer cells induced bone lesions development Radiography revealed larger lesions in mice injected with (**A**, **B**) PC3-ERRα versus PC3-CT, and (**F**, **G**) with PC3c-ERRα versus PC3c-CT. Histology after Goldner's trichrome staining confirmed the radiography results in mice injected with (**C**, **D**) PC3-ERRα versus PC3-CT (**H**, **I**) with PC3c-ERRα versus PC3c-CT (bone matrix in green). (**E**) Induction -of larger bone lesions surface in mice injected with PC3-ERRα (ERRα)(Mann-Whitney, *P* = 0.011), -of a decrease in %BV/TV (Mann-Whitney, *P* = 0.022) and an increase of %TB/STV (Mann-Whitney, *P* = 0.008) compared with mice injected with PC3-CT (CT). Bone formation incidence show that 70% of mice injected with PC3-ERRα (ERRα) developed some bone formation as opposed to 10% of mice injected with PC3-CT (CT). (**J**) Increased -of bone lesions surface in mice injected with PC3c-ERRα (Mann-Whitney, *P* = 0.0175), -of the %BV/TV (Mann-Whitney, *P* = 0.022) and decrease of the %TB/STV (Mann-Whitney, *P* = 0.0023) compared with mice injected with PC3c-CT (CT). Radiography (**K**–**M**) and 3D micro-tomography reconstructions (**N**–**P**) showed larger bone lesions in mice injected with ACE-ERRα versus ACE-CT with an abrogation of the bone lesion effects seen with ERRα overexpression in tumors bearing the dominant negative AF2-truncated ERRα. (**Q**) After 3 weeks post inoculation of ACE-ERRα, ACE-CT and ACE-AF2 cells, radiography revealed larger and smaller bone lesions surface in mice injected with ACE-ERRα and ACE-AF2 respectively compared to CT (Mann-Whitney, *P* = 0.0079, *P* = 0.0304) and microtomographic reconstructions of tibiae show a decrease in mice injected with ACE-ERRα compared to CT (%BV/TV: Mann-Whitney, *P* = 0.0411), an increase in %TB/STV (Wilcoxon, *P* = 0.034) compared to CT, and an increase in % new bone formation/TV (extra-bone spicules formation): (Mann-Whitney, *P* = 0.0025). The increase in bone lesion surface (Mann-Whitney, *P* = 0.0011), in the %TB/STV (Mann-Whitney, *P* = 0.0052), in the extra-bone spicules formation (Mann-Whitney, *P* = 0.0012) and the decrease in %BV/TV (Wilcoxon, *P* = 0.0273) effects seen with ERRα overexpression were markedly abrogated in tumors bearing the dominant negative AF2-truncated ERRα. * = *P* < 0.05; ** = *P* < 0.001; *** = *P* < 0.0001. T: Tumor; * bone formation; Arrow: bone degradation.

To address ERRα function in PCa bone progression, we used three CRPC pre-clinical models, two human models (PC3 and PC3c) and one canine model (ACE-1). Specifically, a full-length *ERR*α cDNA was stably transfected into PC3 cells, which are known for their capacity to form osteolytic lesions *in vivo* [27]. Three independent PC3-ERRα clones (overexpressing *ERRα*) and three PC3-CT clones (harboring empty vector) were generated (Figure 1E, 1F). In parallel, to validate further the human PC3 model, human PC3c and canine ACE-1 PCa cells that both induce mixed bone lesions (with both osteolysis and osteoformation) were stably transfected with full-length *ERR*α cDNA (Figure 1H, 1J) [28] [29]. ACE-1 cells were also transfected with cDNA containing a truncated form of ERRα lacking the co-activator binding domain AF2 (AF2) (Figure 1J) [22]. Western blotting confirmed higher ERRα expression in PC3-ERRα, PC3c-ERRα and ACE-1-ERRα than in their respective control cells (Figure 1E, 1H, 1J). The presence of a slightly lower molecular weight band in AF2 in ACE-1 cells expressing the ERRα-AF2 deletion mutant corresponded well with its expected smaller size (AF2; Figure 1J) [22]. As expected, expression of mRNA for *VEGF-A*, a known *ERRα* target gene [30] was higher in all of the *ERRα* overexpressing clones (*ERRα*; PC3, PC3c and ACE-1) but not in the AF2 ACE-1 clone, confirming the increased activity and the dominant negative functions of both wild-type *ERRα* and the truncated *ERRα-AF2* constructs respectively (Figure 1G, 1I, 1K). To assess whether and how levels of *ERRα* in tumor cells affected progression of bone lesions, PC3, PC3c and ACE-1 clones were inoculated via intra-tibial injections into SCID male mice (Figure 2). Three weeks (for PC3 (pool of the 3 clones for CT and ERRα respectively) and ACE-1 clones) (Figure 2 (PC3 (A–E), ACE-1 (K–Q)) and six weeks (for PC3c clones) (Figure 2 PC3c (F–J)) after tumor cell injections, radiographs revealed that animals bearing *ERRα* overexpressing tumors had increased bone lesion surfaces whereas ACE-AF2 tumors had decreased bone lesion surface compared to CT tumors (Figure 2 -PC3 (A–B), (Mann-Whitney, *P* = 0.011) (bone lesion surface mm^2^)(E), -PC3c (F-G), (Mann-Whitney, *P* = 0.0175)(J) -ACE-1 (K–M) (Mann-Whitney, *P* = 0.0079, *P* = 0.0304) (Q)). The stimulatory effect of ERRα on PCa-induced bone lesion surface was confirmed by three-dimensional micro-computed tomographic reconstruction (%BV/TV) (cortical and trabecular bone), with a decrease in bone volume in animals bearing PC3-ERRα and ACE-1-ERRα tumors (%BV/TV, Mann-Whitney, PC3 *P* = 0.022 and, ACE-1 *P* = 0.0411) suggesting an increase in bone destruction in both ERRα overexpression models (Figure 2E and 2Q, %BV/TV). The stimulatory effect of *ERRα* on PCa-induced bone lesion surface was also evident by histology (Figure 2 PC3(C,D)) and histomorphometric analysis (TB/STV) with an increase of skeletal tumor burden (Figure 2E and 2Q). Since the osteoblastic region is highly stimulated in the PC3c model (Figure 2H, 2I) (see the increased of the %BV/TV: (Mann-Whitney, *P* = 0.022)), the surface of the tumor (TB/STV) decreased in animals bearing PC3c-ERRα (Figure 2J (Mann-Whitney, *P* = 0.0023)) (asterisks showing bone formation). Similarly, 70% of mice bearing PC3-ERRα tumors exhibited small new bone formation compared to mice bearing PC3-CT tumors (Figure 2E). New bone formation was also seen in animals bearing ACE-1-ERRα versus ACE-1-CT tumors (Figure 2Q: extra-bone-new spicules surface formation/ tissue volume; Figure 2 (N-P) (asterisks mark extra-new spicules bone formation). The bone lesion surface and bone volume-new bone formation effects seen with ERRα overexpression were markedly abrogated in tumors bearing the dominant negative AF2-truncated ERRα (Figure 2K, 2M and 2N, 2P, 2Q). Taken together, our results indicate that overexpression of ERRα in PCa cells stimulates both new bone formation and destruction suggesting that it may be associated with mechanisms mediating mixed lesions *in vivo*.

### Modulation of ERRα expression in cancer cells affects the bone microenvironment

**Figure 3:**
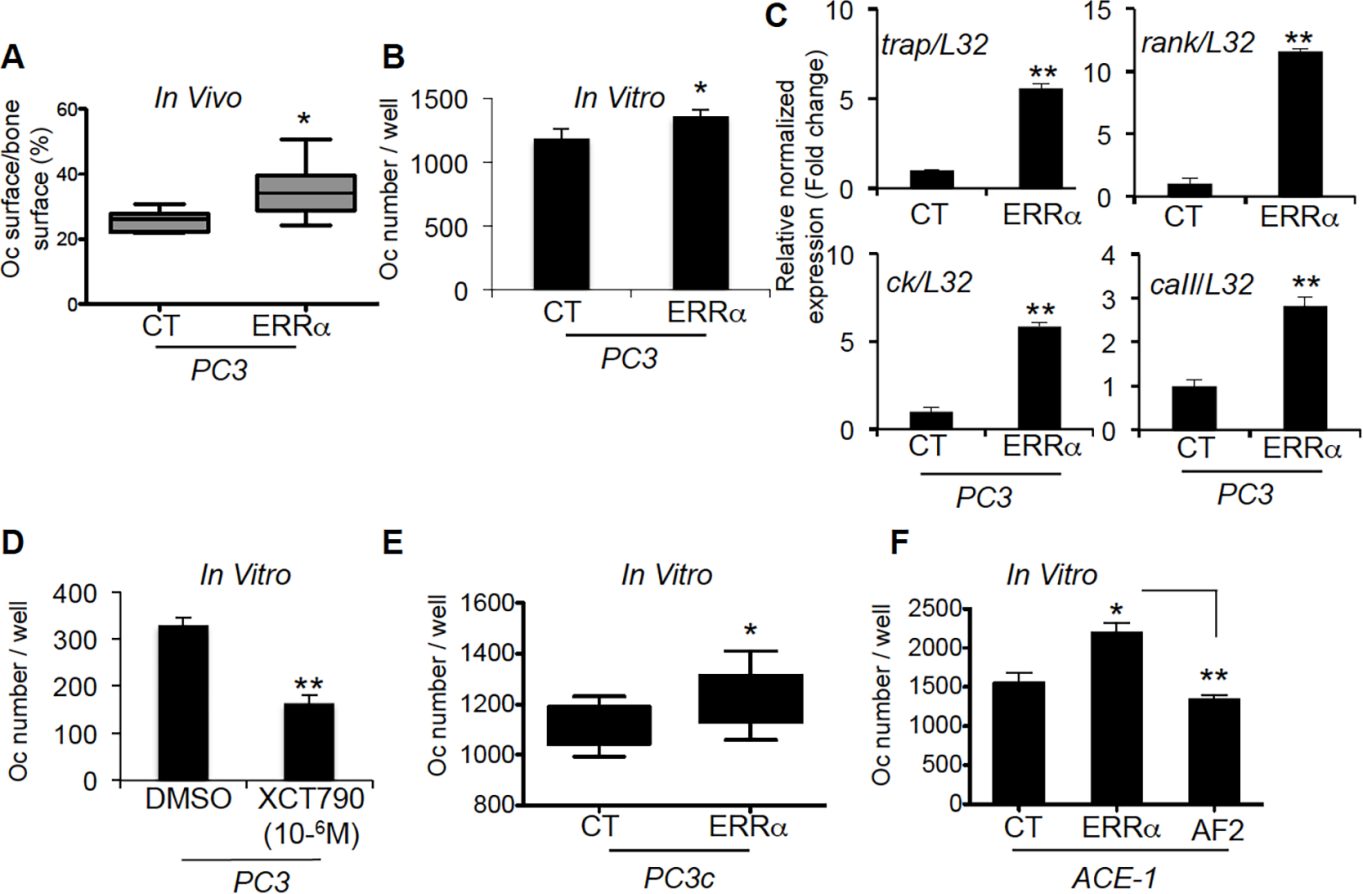
ERRα overexpression in PCa-ERRα cells modified bone-resorbing cells (**A**) Increase in osteoclast (Oc) surface in bone lesions induced by PC3-ERRα cells *in vivo* (%Oc.S/BS: Mann-Whitney, *P* = 0.0062; *n* = 7 (CT) and *n* = 10 (ERRα)). (**B**) PC3-ERRα cells increase the number of TRAP+ osteoclasts *in vitro* (Oc number/well: paired *t*-test, *P* = 0.0275). (**C**) mRNA was extracted from co-cultures on day 7. The expression of *trap* (Tartrate Resistant Acid Phosphatase), *ck* (Cathepsin K), *rank* and *caII* (Carbonic Anhydrase II) was assessed by real-time RT-PCR on triplicate samples; all markers were higher in Oc/PC3-ERRα (ERRα) versus Oc/PC3-CT (Student's *t*-tests, *P* = 0.0026; *P* = 0.0055, *P* = 0.0057; *P* = 0.008). (**D**) Conditioned medium obtained from parental PC3 cells treated with the inverse agonist XCT-790 decreased Oc formation, confirming the results obtained with PC3-ERRα (Student's *t*-tests, *P* = 0.0023). (**E**–**F**) PC3c-ERRα **(**E) and ACE-ERRα (F) increased the number of TRAP+ osteoclasts *in vitro* compared to the respective controls (Oc number/well: paired *t*-test, *P* = 0.022 (PC3c-ERRα versus PC3C-CT) and ANOVA *p* = 0.0048, *P* < 0.05 (ACE-1-ERRα versus ACE-1-CT) while ACE-1-AF2 inhibited Oc formation *P* < 0.005 (ACE-1-AF2 versus ACE-1-ERRα). Oc results are representative of two independent experiments, each performed in triplicate samples.

Since our *in vivo* data suggested an impact of ERRα expression levels on PCa-induced bone destruction and formation, we next assessed whether PCa overexpressing ERRα cells affected osteoclasts (bone-resorbing cells) and osteoblasts (bone-forming cells). A 40% increase in TRAP-positive osteoclast surface (%Oc.S/BS) was seen at the bone-tumor cell interface in PC3-ERRα tumors (Figure 3A). Consistent with these *in vivo* data, the number of TRAP-positive cells (Figure 3B) and the expression of osteoclast markers (*trap*, *ck*, *caII* and *rank*) (Figure 3C) were higher in co-cultures of primary mouse bone marrow cells with PC3-ERRα cells compared to PC3-CT cells [5]. Moreover, treatment of bone marrow cells by the conditioned medium obtained from parental PC3 cells treated with the inverse agonist XCT-790, which blocks ERRα activity, inhibited osteoclast formation (Figure 3D). Similarly, PC3c- ERRα cells co-cultured with primary mouse bone marrow cells also stimulated osteoclast formation compared to PC3c-CT cells (Figure 3E), as did ACE-1-ERRα compared to ACE-1-CT cells while ACE-1-AF2 inhibited osteoclastogenesis compared to ACE-1-ERRα (Figure 3F) suggesting that cancerous cells expressing ERRα increase osteoclastogenesis.

**Figure 4:**
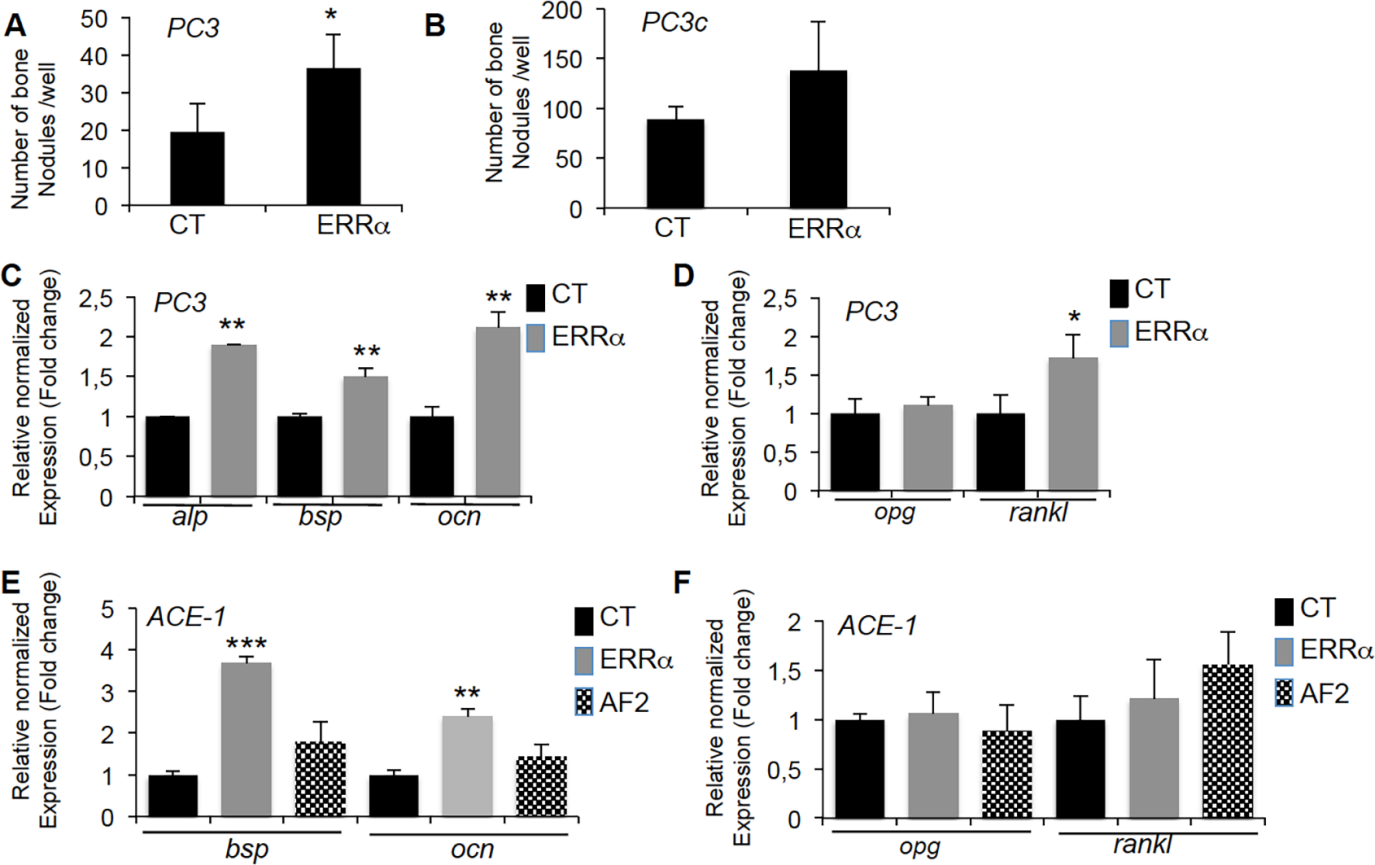
ERRα overexpression in PCa-ERRα cells stimulates bone-forming cells (**A**) Osteoblasts (Ob) from primary mouse calvaria cell cultures were treated with conditioned medium of PC3-ERRα (ERRα) or PC3-CT (CT) cells. Bone nodule number was higher in co-cultures with PC3-overexpressing ERRα (Ob-ERRα) (Mann-Whitney,*P* = 0.0118). (**B**) When treated with conditioned medium of PC3c-ERRα (ERRα), bone nodule number was increased but not significantly compared to PC3c-CT (CT). (**C**) Confirming mouse calvaria results, co-cultures of PC3-ERRα (ERRα) or PC3-CT (CT) cells with the MC3T3-E1 osteoblastic cell line for 2 weeks stimulated expression of osteoblastic markers alp (Alcalin Phosphatase), bsp (bone sialoprotein) and ocn (osteocalcin) (ANOVA, *P* < 0.0001; Student's *t*-tests, *P* = 0.0031; *P* = 0.0098; *P* = 0.0017) (**D**) Expression of rankl mRNA was also higher (ANOVA, *P* < 0.0001, Student's *t*-tests, *P* = 0.0279) while opg expression was not altered. (**E**) Co-cultures of ACE-1-ERRα (ERRα) or ACE-1-CT (CT) cells with the MC3T3-E1 osteoblastic cell line for 2 weeks also stimulated bsp and ocn mRNA expression (ANOVA bsp, P = 0.004; Student's *t*-tests, P = 0.0003 (ACE-1-CT versus ACE-1-ERRα); ANOVA ocn, *P* = 0.0069; Student's *t*-tests, *P* = 0.0067 (ACE-1-CT versus ACE-1-ERRα) while no regulation was observed with ACE-1-AF2 cells (AF2). (**F**) Expression of rankl and opg mRNA was not regulated. Ob results are representative of two independent experiments, each performed in triplicate samples.

The increased bone formation observed *in vivo* suggests that changes in *ERRα* expression in PCa cells also alters the differentiation of osteoblasts. Consistent with this hypothesis, a higher number of bone nodules formed in primary mouse calvaria cells cultured with PC3-ERRα versus PC3-CT conditioned medium (Figure 4A). Similarly, the expression of the osteoblastic markers alkaline phosphatase (*alp*), bone sialoprotein (*bsp*) and osteocalcin (*ocn*) increased (Figure 4C) in co-cultures of MC3T3-E1 and PC3-ERRα cells (ERRα) (Figure 4C) [31]. The pro-osteoclastic factors *rankl* but not *opg*, was increased in MC3T3-E1 cells co-cultured with PC3-ERRα cells (Figure 4D). Consistent with this, we observed a trend towards an increase in the number of bone nodules formed in primary mouse calvaria cells cultured with PC3c-ERRα conditioned medium (Figure 4B) and an increase in osteoblastic markers *bsp* and *ocn* expression in co-cultures of MC3T3-E1 and ACE-1-ERRα cells (ERRα) (Figure 4E). In contrast, no change in expression of either osteoblastic markers was detected in co-cultures of MC3T3-E1 with ACE-1-AF2 cells (Figure 4E). Also no regulation of *rankl* and *opg* expression was noticed (Figure 4F). Taken together, our results suggest that ERRα expression in PCa cells induces changes in osteoblast lineage cells.

### Identification of two factors regulated in PCa cells that couple bone resorption and formation: VEGFA and Wnt5a

**Figure 5:**
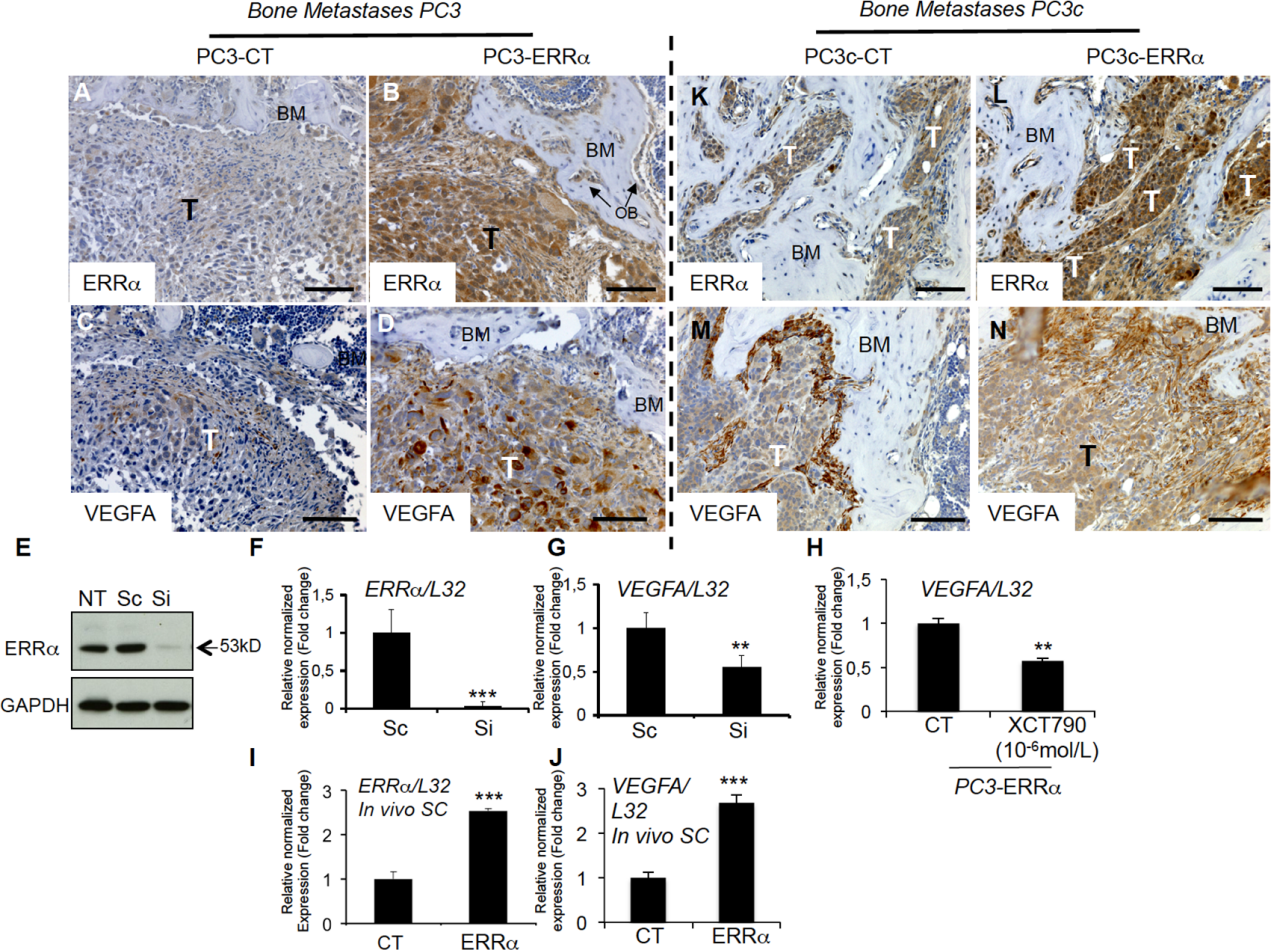
Stimulation of VEGF-A expression in PCa cells by ERRα (**A**–**B**) Visualization of the overexpression of *ERRα* protein expression in tumor by IHC on bone lesions *in vivo* induced by PC3-ERRα (ERRα)(B) compared PC3-CT (CT)(A). (**C**–**D**) Paralleling the overexpression of ERRα, VEGF-A expression in tumor was also stimulated *in vivo* in bone lesions induced by PC3-ERRα (ERRα) (D) compared PC3-CT (CT) cells (C). (**E**) Decreased ERRα protein expression by transfection of three pooled siRNA sequences in parental PC3 cells shown by Western blot and (**F**–**G**) by real-time RT-PCR for *ERRα* and *VEGF-A* expression (Student's *t*-tests *P* = 0.0002; *P* = 0.0027). (**H**) Decreased *VEGF-A* mRNA expression was also observed after XCT-790 treatment at 10^−6^M for 48 h in PC3-ERRα cells (Student's *t*-tests *P* = 0.006). (**I**–**J**) Real-time RT-PCR was performed on triplicate samples and normalized against the ribosomal protein gene *L32* to evaluate ERRα (*P* = 0.0001) and VEGF-A (*P* = 0.0001) expression within subcutaneous (SC) tumors *in vivo* (pool of *n* = 3 for each condition). (**K–N**) Similarly, to the PC3 model, IHC revealed that *ERRα* and *VEGF-A* protein levels in tumors were increased in mixed lesions induced by PC3c-ERRα (ERRα) (L and N concomitantly) compared to PC3c-CT (CT) (K and M concomitantly) *in vivo*. Bar = 200 μm, T: Tumor; OB: osteoblasts; BM: Bone Matrix.

Since both osteoclasts and osteoblasts are stimulated by PCa cells overexpressing ERRα, we next sought to identify factors secreted by PCa cells that may stimulate both lineages. As shown earlier, PC3, PC3c and ACE-1 cells overexpressing *ERRα* stimulate expression of *VEGF-A*(Figure 1G, I, K), a known ERRα target and stimulator of osteoblasts and osteoclasts [32, 33]. By immunohistochemistry, *ERRα* (Figure 5A, 5B) and *VEGF-A* (Figure 5C, 5D) expression was observed in PCa cells and higher in PC3-ERRα-induced bone lesions *in vivo* (Figure 5B and 5D respectively) compared to PC3-CT lesions (Figure 5A and 5C respectively). Regulation of *VEGF-A* by ERRα was confirmed *in vitro* in PC3 cells transfected with a pool of three ERRα-siRNA sequences (Si) compared to control sequences (Sc) (Figure 5E–5G). Blocking ERRα activity by treating PC3-ERRα cells with the ERRα inverse agonist XCT-790 also demonstrated a statistical down-regulation of *VEGF-A* (Figure 5H). That ERRα regulates *VEGF-A* in both non-bone and bone microenvironments was determined by assessing subcutaneous (SC) tumors obtained by injecting PC3-ERRα and PC3-CT clones in male SCID mice. *VEGF-A* expression was higher in PC3-ERRα (ERRα) -tumors compared to PC3-CT (CT)-induced SC tumors *in vivo* (Figure 5I–5J). The correlation of increased *ERRα* and increased *VEGF-A* expression was confirmed in PC3c-ERRα-induced bone lesions *in vivo* (Figure 5L, 5N) compared to PC3c-CT lesions (Figure 5K, 5M). It is interesting to mention that beside tumor mass, *vegf-a* was also highly expressed in peri-tumoral cells in PC3c-CT and PC3c-ERRα (Figure 5M, 5N) compared to PC3-CT and PC3-ERRα- induced bone lesions *in vivo* (Figure 5C, 5D). This peri-tumoral *vegf-a* expression that probably contributes to the formation of the mixed phenotype observed in PC3c cells, was not modulated in PC3-ERRα- induced bone lesions *in vivo* compared to CT. Finally, in line with our pre-clinical models, we found that *VEGF-A* positively correlated with *ERRα* expression levels in primary CRPC tumor samples from combined microarray datasets GSE1034, GSE6919 and GSE2269 (*n* = 140, *r* = 0.364, *P* < 0.0001) and in bone metastases (GSE29650, GSE41619 and GSE74685) [34–36] (*n* = 64, *r* = 0.646, *P* < 0.0001) (Table 1; Supplementary Table S1).

**Table 1:** Meta-analysis using public datasets showing correlation between *ERRα* and *VEGFA*, *WNT5A*, *POSTN* and *TGFb1* expression levels in bone metastases (GSE29650, GSE41619 and GSE74685) and/or in primary CRPC tumor (GSE1034, GSE6919 and GSE2269)

			Number of samples (N)	
Genes names	Database	Correlations	Primary tumour	Bone Metastases
VEGFA/ERRA	GSE21034/ GSE6919/ GSE32269	Pearson R = 0.364 *P* = 0.00001	140 CRPC	N/A
	GSE29650/ GSE41619/GSE74685	Pearson R = 0.646 *P* = 0.000001	N/A	64
WNT5A/ERRA	GSE21034/ GSE6919/ GSE32269	Pearson R =−0.178 *P* = 0.035	140 CRPC	N/A
	GSE29650/ GSE41619/GSE74685	Pearson R = 0.342 *P* = 0.00572	N/A	64
POSTN/ERRA	GSE21034/ GSE6919/ GSE32269	Pearson R = 0.401 *P* = 0.000001	184 in adjacent normal tissue	N/A
	GSE29650/ GSE41619	Pearson R = 0.528 *P* = 0.00023	N/A	44
TGFb1/ERRA	GSE21034/ GSE6919/ GSE32269	Pearson R = 0.684 *P* = 0.000001	140 CRPC	N/A
	GSE29650/ GSE41619	Pearson R =−0.365 NS	N/A	14

To obtain z-scores, normalized data were log_2_ transformed and calculated by subtracting the population mean from individual expression values for each gene and then dividing the difference by the population standard deviation. Correlation scores were calculated using the Pearson correlation coefficient. *P*-values less than 0.05 were considered statistically significant.

**Figure 6:**
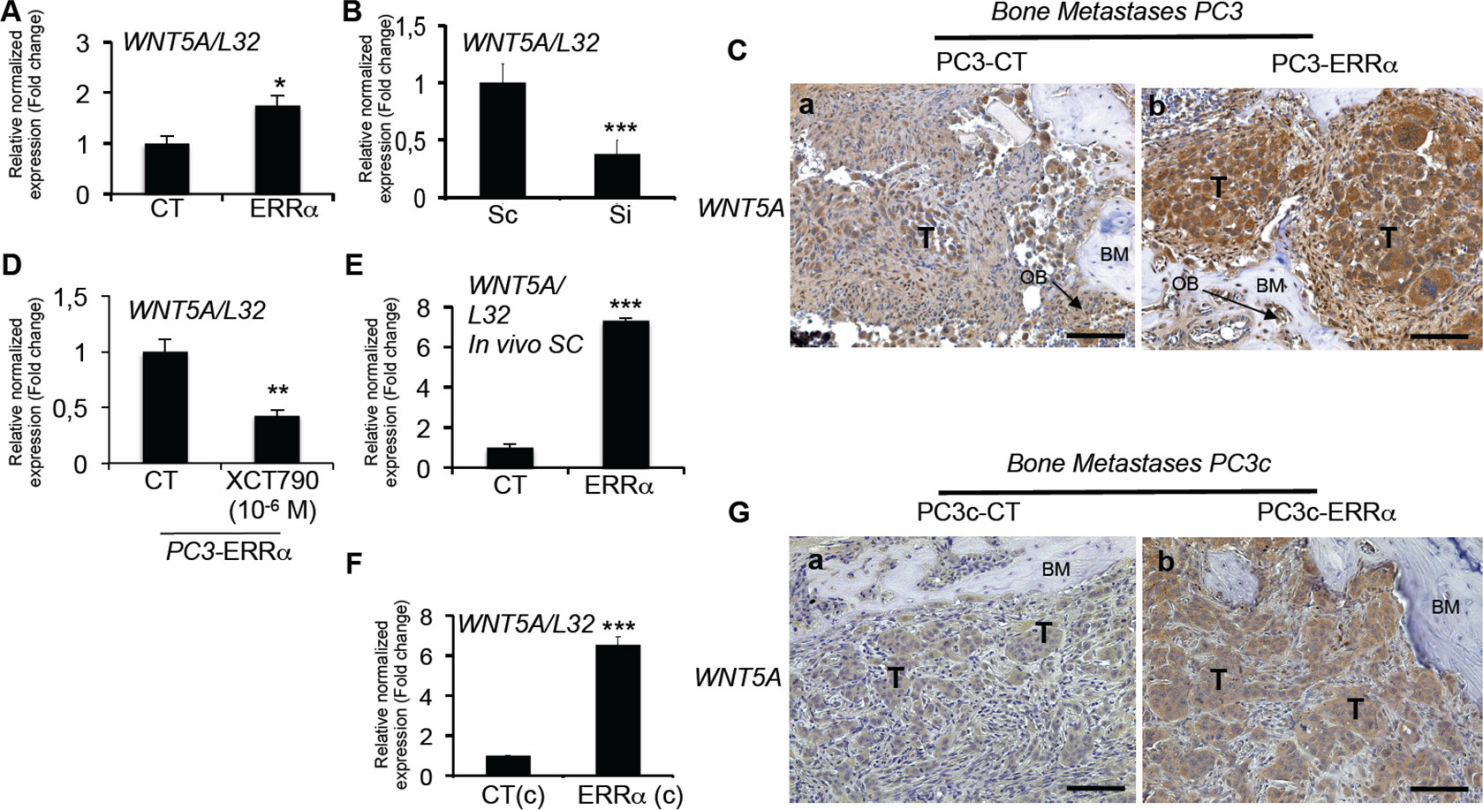
Stimulation of WNT5A expression in PCa cells by ERRα (**A**) Assessment of *WNT5A* expression by real-time RT-PCR on triplicate samples and normalized against the ribosomal protein gene *L32. WNT5A* (Student's *t*-tests *P* = 0.0305) was higher in PC3-ERRα versus PC3-CT (CT) cells *in vitro*. (**B**) *WNT5A* (Student's *t*-tests *P*= 0.0007) mRNA expression was decreased in Si-ERRα (pooled of three SiRNA sequences) transfected cells compared to control-transfected (Sc) cells. (**C**) Paralleling the overexpression of ERRα, *WNT5A* protein level expression was also stimulated in tumor *in vivo* in bone lesions induced by PC3-ERRα (ERRα)(b) compared PC3-CT (CT) cells (a). (**D**) Decreased *WNT5A* mRNA expression was also observed after XCT-790 treatment at 10^−6^M for 48 h in PC3-ERRα cells (Student's *t*-tests *P* = 0.0077). (**E**) By real-time RT-PCR, we also found *WNT5A* mRNA expression (Student's *t*-tests *P* = 0.0001) increased in PC3-ERRα SC tumors *in vivo* compared to PC3-CT SC tumors (**F**) *WNT5A* (Student's *t*-tests *P* = 0.0026) mRNA expression was also upregulated in the PC3c-ERRα ((ERRα(c)) clone compared to the PC3c-CT (CT(c)) *in vitro* and (**G**) in tumor *in vivo* in bone lesions induced by PC3c-ERRα (ERRα)(b) compared PC3c-CT (CT) cells (a). Bar = 200 μm, T: Tumor; OB: osteoblasts; BM: Bone Matrix.

The second factor that was found upregulated by ERRα in our human preclinical models is *WNT5A*, a factor also known to stimulate osteoblasts and osteoclasts [37] and previously linked to ERRα in osteoblasts [38]. Thus, *WNT5A* expression was higher in ERRα-overexpressing PC3 cells (PC3-ERRα) compared to PC3-CT clones (Figure 6A), and down-regulating ERRα levels in PC3 cells with ERRα-SiRNA (pool of three sequences) decreased *WNT5A* expression compared to control sequences (Sc, Figure 6B). *WNT5A* expression was also upregulated in PC3-ERRα− versus PC3-CT-induced bone lesions *in vivo* (Figure 6C, a versus b). XCT-790 treated PC3-ERRα cells also demonstrated a down-regulation of *WNT5A* expression (Figure 6D). Stimulation of *WNT5A* expression by ERRα was also confirmed in PC3-ERRα− versus PC3-CT-induced SC tumors *in vivo* (Figure 6E), and in PC3c-ERRα-versus PC3c-CT clones *in vitro* (Figure 6F) as well as in PC3c-ERRα versus PC3c-CT-induced bone lesions *in vivo* (Figure 6G, a versus b). Notably, *WNT5A* expression also positively correlated with *ERRα* expression levels in bone metastases microarray datasets (GSE29650, GSE41619 and GSE74685) (*n* = 64, *r* = 0.342, *P* = 0.00572). Contrary to bone metastases specimens, a negative correlation with a weak Pearson R value was observed in primary CRPC tumor samples from combined microarray datasets GSE1034, GSE6919 and GSE2269 (*n* = 140, *r* = −0.178, *P* < 0.0001) (Table 1; Supplementary Table S1).

### Stimulation of stromal POSTN expression by tumor cells overexpressing ERRα

**Figure 7:**
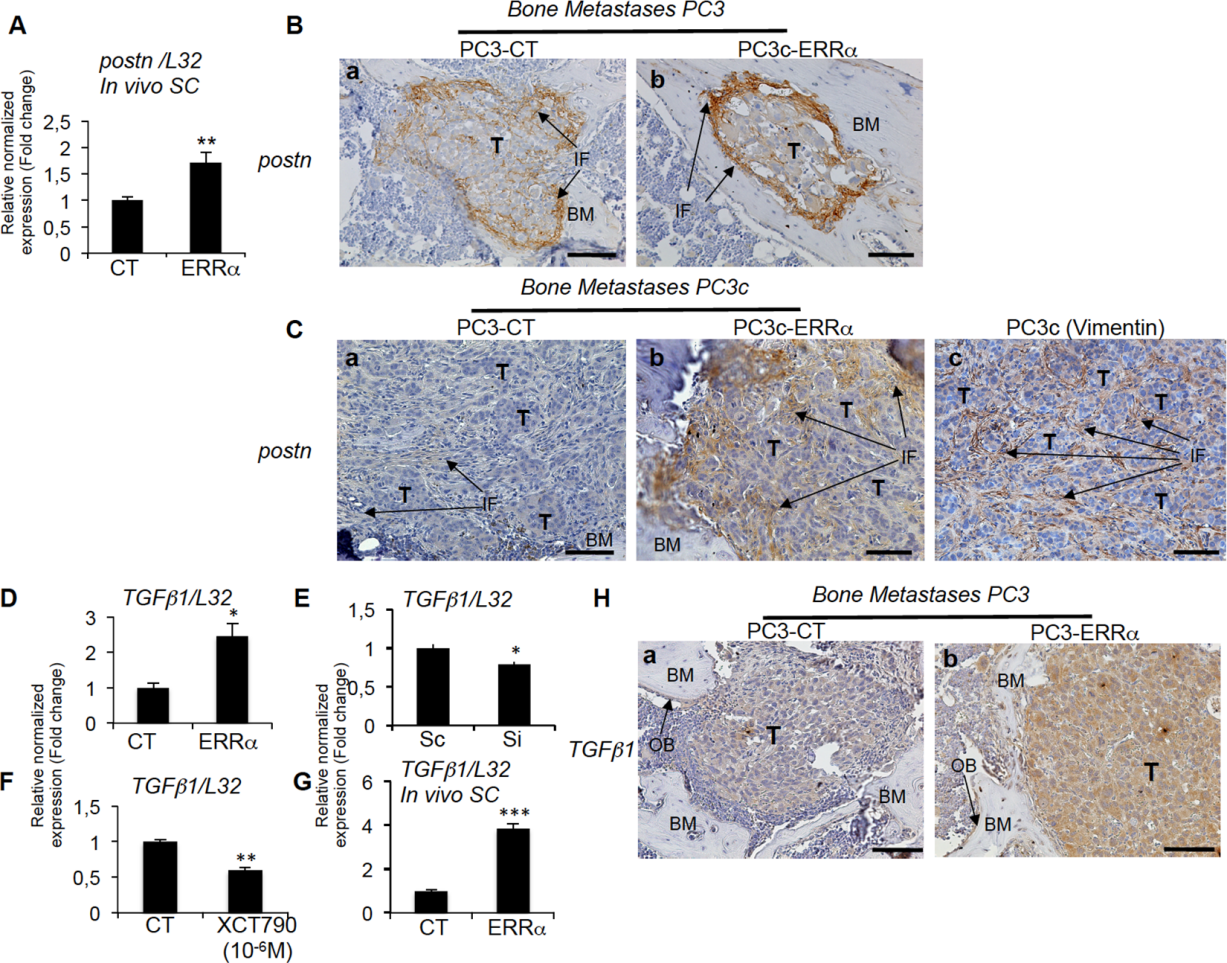
Regulation of stromal postn by ERRα (**A**) Assessment of the mouse *postn* expression by real-time RT-PCR on triplicate samples and normalized against the mouse ribosomal protein gene *L32.* Real-time RT-PCR showed increased *postn* mRNA expression (Student's *t*-tests *P* = 0.0026) in PC3-ERRα-induced SC tumors *in vivo*. (**B**) Increased expression of *postn* in peri-tumoral and infiltrating fibroblasts was visualized by IHC on PC3-ERRα-induced bone lesions (b) compared to PC3-CT (a). (**C**) Similarly, *postn* protein was upregulated in the infiltrating fibroblasts *in vivo* in bone lesions induced by PC3c-ERRα (ERRα)(b) compared PC3c-CT (CT) cells (a). Vimentin antibody was used to show the fibroblastic type of the *postn* expressing cells (c) (**D**) Real-time RT-PCR showed that *TGFb1* mRNA expression (Student's *t*-tests *P* = 0.0246) was higher in PC3-ERRα versus PC3-CT (CT) cells *in vitro* while (**E**) *TGFb1* (Student's *t*-tests *P* = 0.022) mRNA expression was decreased in Si-ERRα (pooled of three sequences) transfected cells compared to control-transfected (Sc) cells. (**F**) Real-time RT-PCR also showed decreased *TGFb1* mRNA expression (Student's *t*-tests *P* = 0.0033) in XCT790 treated PC3-ERRα cells and (**G**) increased *TGFb1* mRNA expression (Student's *t*-tests *P* = 0.0002) in PC3-ERRα-induced SC tumors *in vivo*. (**H**) *TGFb1* staining was higher in PC3-ERRα (b) compared to PC3-CT (a) in tumor-induced bone lesions *in vivo*. Bar = 200 μm. T: Tumor; OB: osteoblasts; BM: Bone Matrix; IF: infiltrating fibroblasts.

Given that WNT signaling is known to be recruited by the extracellular matrix protein PERIOSTINE (*POSTN*) in the stroma of at least certain tumor types and to participate in the steps required for cancer cell colonization and tumor progression [8], we next analyzed *postn* expression in our tumor models. By real time PCR, we found mouse *postn* expression up-regulated in PC3-ERRα versus PC3-CT SC tumors (Figure 7A). Immunostaining confirmed the stimulation of *postn* in peri-tumoral infiltrating cells in PC3-ERRα bone lesions (Figure 7B, a versus b), in PC3c-ERRα bone lesions (Figure 7C, a versus b) and in peri-tumoral cells in PC3-ERRα SC tumor versus PC3-CT SC tumors (Supplementary Figure S1A a versus b). Vimentin expression was also addressed to show the stromal phenotype of the peri-tumoral cells expressing *postn* (Figure 7Cc). Importantly, *ERRα* and *postn* expression levels were significantly positively correlated in bone metastases specimens (*n* = 44, *r* = 0.528, *P* = 0.00023) (Table 1; Supplementary Table S1). A positive correlation was also found in adjacent normal tissue of primary CRPC tumors (*n* = 184, *r* = 0.401, *P* = < 0.0001) confirming the stimulation of *POSTN* in peri-tumoral cells (Table1; Supplementary Table S1).

*Postn* has been shown to be stimulated in infiltrating stromal cells by factors secreted by breast cancer cells such as TGFβ [8, 9]. By real-time PCR, we found higher human *TGFb1* expression in PC3-ERRα clones compared to PC3-CT (Figure 7D). Regulation of *TGFb1* expression by *ERRα* in PCa cells was also confirmed by the lower levels of *TGFb1* expression in ERRα-SiRNA versus control sequences-transfected PC3 cells (Si versus Sc, Figure 7E), in XCT-790 treated PC3-ERRα cells versus DMSO (Figure 7F) and by its stimulation in PC3-ERRα versus PC3-CT SC tumors *in vivo* (Figure 7G). Immunohistochemistry also confirmed higher *TGFb1* staining in tumor cells in PC3-ERRα (Figure 7Hb) compared to PC3-CT-induced (Figure 7Ha) bone lesions *in vivo* and in PC3c-ERRα (Supplementary Figure S1Bb) compared to PC3c-CT-induced bone lesions *in vivo* (Supplementary Figure S1Ba). Finally, *ERR*α and *TGFb1* expression levels were significantly positively correlated in primary CRPC tumor samples (*n* = 140, *r* = 0.684, *P* = < 0.0001) (Table 1; Supplementary Table S1). On the contrary, no significant correlation was observed in bone metastases (Table 1; Supplementary Table S1). The data suggest that the upregulation of stromal cell *POSTN* may be the initial step through which ERRα induces rapid bone lesion progression.

## DISCUSSION

Our findings indicate that ERRα is a stimulator of prostate cancer cell progression in bone. In addition to confirming the regulation of the previously identified ERRα-regulated gene, *VEGF-A* [30] we identified *WNT5A* and *TGFb1* as new ERRα-regulated genes. Together with our observation that ERRα expression is higher in CRPC patients with bone metastases than without, and that ERRα in PCa alters molecular signaling in the stroma via the regulation of POSTN expression in infiltrating fibroblasts [10], our data provide new mechanistic insights into ERRα-regulated pathways mediating PCa progression in bone.

ERRα-positive prostate tumors have been associated with more invasive disease and a higher risk of recurrence [18]. For example, *ERRα* immunoreactivity score has been linked with Gleason scores [18]. ERRα was also recently associated with hypoxia adaptation in PCa cells (AR+ (LNCaP) and AR-(PC3) pre-clinical models), suggesting that *ERRα* might be involved in advanced progression of PCa [19]. Advanced PCa has a propensity of 90% to metastasize to bone, of 25% to metastasize to liver and to develop CRPC, all of which are associated with poor five-year survival rate [2].

Since we had shown that ERRα may be involved in bone metastases from breast cancer [22] that prompted us to determine if and how ERRα could contribute to malignant growth of PCa in bone. Interestingly, ERRα stimulation of PCa-induced bone lesion progression is different from what we observed in breast cancer [22]. ERRα plays a dual role in the progression of breast cancer -by promoting local tumor growth but by decreasing osteolytic lesions in bone, the latter due to the inhibition of osteoclast differentiation. The opposite activity of ERRα in PCa versus breast cancer on bone resorbing-cells may explain the differences in tumor progression in bone between breast and prostate cancers [22] and suggests distinct clinical management of prostate versus breast cancer patients with bone metastases.

**Figure 8:**
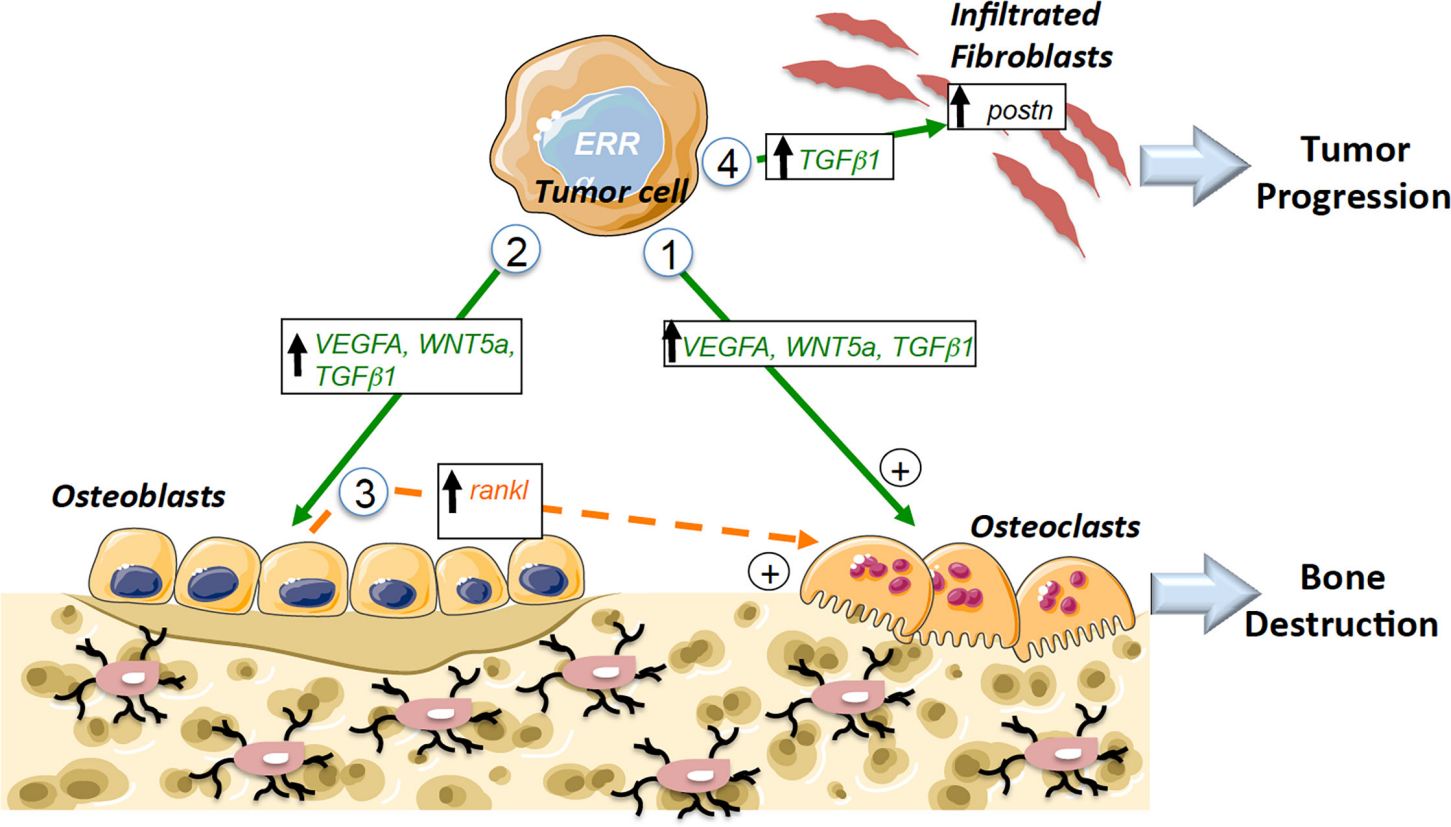
Schematic diagram showing ERRα pathways that can mediate tumor progression in bone Pathway 1: Increased expression of pro-resorption factors (*VEGF-A*, *WNT5A*, *TGF*β*1*) in PCa tumors overexpressing *ERRα* leads to direct stimulation of Oc formation. Pathway 2: Increased expression of pro-osteoblastic factors (*VEGF-A*,*WNT5A*, *TGF*β*1*) in PCa tumors overexpressing ERRα that leads to direct stimulation of Ob formation. Pathway 3: Indirect stimulation of osteoclasts through osteoblasts (via the regulation of *rankl* in Ob for the osteolytic model). Pathway 4: Stimulation of the metastatic niche through the stimulation of *postn* in fibroblasts infiltrating tumors by *TGF*β*1* secreted by tumor cells. Ob: osteoblast; Oc: osteoclast; direct signaling (in green), indirect signaling (in orange).

ERRα expression levels in primary tumors and bone metastases associated with PCa patients correlated well with our results in three CRPC preclinical mouse models. At the cellular level, we found that ERRα directly stimulates both osteoclasts and osteoblasts, as well as factors in PCa cells thought to stimulate osteoclasts and osteoblasts, *i.e.*, *VEGF-A* [33] [39]; *WNT5A* [37, 40, 41] and *TGFb1* [1, 6, 42] (Figure 8, pathways 1 and 2). Notably, all these factors were also found to correlate with *ERRα* expression in our meta-analysis studies of human PCa datasets.

Previously, *WNT11* was shown to be regulated by *ERRα* in PCa cells and implicated in cancer cell invasion [43]. Our data on *WNT5A* and *ERRα* expression in both *in vivo* and *in vitro* experiments and meta-analysis of human PCa datasets reinforces the link between *ERRα* activity and Wnt signaling. Interestingly, analysis of circulating tumor cells (CTC) from patients had indicate that *WNT5A* is activated, in keeping with a role for *WNT5A* in cell polarization and cancer cell invasion [44, 45, 46]. Indeed, analysis of CTCs from patients progressing on AR inhibitor compared with untreated case indicates activation of *WNT5A* [46]. Moreover, Miyamoto D *et al.* show that ectopic expression of *WNT5A* inhibits the anti-proliferative effect of AR inhibition suggesting that it may participate in the acquisition of resistance to androgen deprivation and to treatment failure [46]. However, *WNT5A* has variously been described as a pro or an inhibitor of tumor growth in bone [47, 48]. Our results suggest that ERRα-mediated *WNT5A* is associated with advanced PCa metastatic disease in bone, but to fully understand the molecular mechanisms and determine direct versus indirect regulation by *ERRα* in tumor cells, additional studies are required. Nevertheless, the identification of *WNT5A* as an ERRα-regulated gene may represent implications for its potential use as a prognostic marker in prostate cancer.

With respect to PCa-induced osteolysis, we found that *rankl* expression was stimulated in MC3T3-E1 cells co-cultured with PC3-ERRα but not with the ACE-1 cell model, which is a mixed lesion model, whether or not ERRα levels were modified in the latter. These data suggest indirect regulatory effects on osteoclasts that participate in the resorption observed in bone lesions in animals bearing ERRα osteolytic tumors but not mixed lesions (Figure 8, pathway 3).

Our data suggest that ERRα may also have an impact on the stromal microenvironment. The reciprocal interaction between cancer cells and tissue-specific stroma is known to be critical for primary and metastatic tumor growth progression and *POSTN* is one of the extracellular matrix molecules known to participate in tumor proliferation and invasion when secreted from stromal cells infiltrating tumors [8, 10, 11]. Here, we confirm that *postn* is not only expressed in the stromal cells infiltrating tumors of bone xenografts but also in orthotopic PCa xenografts [10]. However, we also show for the first time that mouse *postn* expression can be stimulated by PCa cells overexpressing ERRα in bone and in SC tumors *in vivo*. Our data also suggest that ERRα may participate in the initial expansion of cancer cells by regulating the extracellular matrix component *postn* in stromal cells infiltrating tumors at least in part through the regulation of *TGFb1* in prostate cancer cells (Figure 8, pathway 4) [8]. The link between *ERRα/POSTN* in the clinical models was confirmed by our meta-analyses of bone metastases and in tumor adjacent tissues in primary tumors, which suggests that *POSTN* may constitute a new prognostic marker in association with ERRα. The data also suggest that this is may be the initial step by which ERRα induces rapid bone lesion progression. Consequently, targeting the tumor microenvironment may also constitute a new therapeutic approach for prostate cancer patients with high levels of ERRα [49]

Very recently, the ERRα coactivator PGC1α has been described as a suppressor of PCa metastasis [50]. PGC1α is expressed at very low levels (*i.e.*, CT: 33) in our PC3, PC3c models, suggesting that the ERRα-PGC1α mechanisms described by Torrano *et al.* are unlikely to occur in these models. Taken together, these data suggest that ERRα is a suppressor when PGC1α is highly expressed and as an activator when PGC1α expression is low, in turn strongly suggesting that, depending on the expression of ERRα cofactors in PCa cells, ERRα can have opposite activities. Thus, it will be important to take into account not only the expression of ERRα but also its cofactors in tumors specimens.

In conclusion, we report for the first time on the involvement of ERRα in the steps of PCa initiation and tumor progression in bone, which results from ERRα modifications of signaling molecules and the microenvironment. Our findings contribute to a new understanding of mechanisms underlying the overall negative clinical outcome associated with ERRα in prostate cancer and provide a rationale for the investigation of ERRα as a therapeutic target to treat primary prostate cancers and their associated bone metastases.

## MATERIALS AND METHODS

### Ethics approval

SCID male mice were purchased from Charles River and handled according to the French Ministerial Decree No.87-848 of 19 October 1987. Experimental protocols were approved by the Institutional Animal Care and Use Committee at the Université Lyon-1 (France) (CEEA-55 Comité d'Ethique en Expérimentation Animale DR2014-32). Studies involving human primary prostate tumors were carried out according to the principles embodied in the Declaration of Helsinki.

### PCa patient primary and bone metastases specimens for IHC

Autopsy files (dating from 1991 to 1998) from the Department of Pathology (Tumor Tissue Bank, Centre Hospitalier Universitaire de Liège, Belgium) were screened for diagnosis of disseminated prostate cancer with histologically proven bone metastases [51]. For immunohistochemistry (IHC) analysis, we selected a prostate cancer patient who died with disseminated disease, including bone metastases. We retrieved patient slides and their clinical history. Tumors were fixed and paraffin-embedded.

### Meta-analysis

For correlation analysis in clinical samples, gene expression data and corresponding clinical annotations for previously published datasets on normal prostate tissue, adjacent prostate tissue, primary and bone metastases were downloaded from the Gene-Expression-Omnibus (GSE6919-GSE29650-GSE32269-GSE41619-GSE21034-GSE74685) (Supplementary Table S1). To obtain z-scores, normalized data were log_2_ transformed and calculated by subtracting the population mean from individual expression values for each gene and then dividing the difference by the population standard deviation. Correlation scores were calculated using the Pearson correlation coefficient. *P*-values less than 0.05 were considered statistically significant. Statistical significance was determined by GraphPad Prism v 5.02 using the two-sided student *t*-test.

### Animal studies

SCID male mice, 6 weeks of age, were housed in barrier conditions under isolated laminar flow hoods. Mice bearing tumor xenografts were closely monitored for established signs of distress and discomfort and were humanely euthanized.

PC3 cells induce osteolytic lesions, PC3c cells induce mixed lesions with lytic and osteoblastic regions in the bone marrow cavity [28] while ACE-1 mainly induce lytic lesions in the bone marrow cavity and osteoblastic region outside the bone (spicules) [29].

Intra-osseous tumor xenograft experiments were performed as previously described [28] as for technical reason, we could not obtain bone lesions after intra-arterial and intra-cardiac injections. PC3, PC3c and ACE-1 cells (6 × 10^5^ in 15μL PBS) were injected in the bone marrow cavity [28]. Mice were sacrificed after 3 weeks (PC3 and ACE-1) and 6 weeks (PC3c). Radiographs (LifeRay HM Plus, Ferrania) of animals were taken at that time after inoculation using X-ray (MX-20; Faxitron X-ray Corporation). Animals were sacrificed, hind limbs were collected for histology and histomorphometrics analyses. The bone lesion surface, that includes lytic and osteoblastic regions, was measured using the computerized image analysis system MorphoExpert (Exploranova). The extent of bone lesions for each animal was expressed in mm^2^. Tibiae were scanned using microcomputed tomography (Skyscan1174, Skyscan) with an 8.1 μM voxel size and an X-ray tube (50 kV; 80 μA) with 0.5 μm aluminum filter. Three-dimensional reconstructions and rendering were performed using the manufacturer's suite (Respectively, NRecon&CTVox, and Skyscan). Bone Volume/Tissue Volume: (%BV/TV) includes residual trabecular and remaining cortical bone for PC3 and ACE-1 models, plus new bone formation into the bone marrow cavity for PC3c model. For the ACE-1 model, since the osteoblastic lesions is outside the cortical bone, the percent (%) of extra-new bone spicules formation (only spicules surface) with respect to total volume (trabecular, cortical and spicule) (for the ACE model) were quantified. %BV/TV and % of extra-new bone spicules formation were carried out with CTAn (version 1.9, Skyscan) and CTVol (version 2.0, Skyscan) software. Dissected bones were then processed for histological and histomorphometric analysis (tumor burden-to-soft tissue volume (%TB/STV)) and TRAP-positive trabecular bone surface (Oc.S) to the total bone surface (BS) as described previously for the PC3 model [22]. Results were plotted as the mean ± SD.

Subcutaneous injections of PC3 cells (10^6^ in 100 μl PBS) were also performed in 6- to 8-week-old SCID mice. Mice were sacrificed after 6 weeks and tumors were fixed and embedded in paraffin.

### Cell lines and transfection

The PC3 cell line was obtained from the American Type Culture Collection (ATCC) (cell line authentication was performed). The PC3c cells, a subculture cell line of PC3 was isolated in our laboratory *in vitro* after single cell population culture. Consequently to spontaneous derivation of the cells, we finally obtained a subculture cell line named PC3c which was chosen based on its epithelial phenotype [28]. Osteoprotegerin (OPG) and endothelin-1 (ET1) are highly expressed by PC3c while dikkopf-1 (DKK1) expression is decreased. Finally, PC3c highly expressed bone associated markers osteopontin, Runx2, alkalin phosphatase, bone sialoprotein and produced mineralized matrix *in vitro* in osteogenic conditions showing a strong osteomimetism phenotype of PC3c cells compared to PC3 [28]. ACE-1 were obtained from Dr T Rosol [29]. PC3 and ACE-1 stably transfected clones were routinely cultured in F12K (Gibco/Life Technologies) and PC3c clones were cultured in DMEM. All cultures were supplemented with 10% (v/v) fetal bovine serum (Perbio/Thermo Scientific) and 1% (v/v) penicillin/streptomycin (Life technologies) at 37°C in a 5% CO_2_ incubator.

Human *ERR*α cDNA (*ERR*α) and the dominant negative form mutated into the co-activator domain AF2 (AF2) was obtained previously [22]. Cells were cultured under clonal selection for 4 weeks in the presence of puromycin (1 μg/mL) (Life Technologies). PC3 cells were also transfected (100 pmol) with ERRα-siRNA sequences specific to human (ON-TARGET plus Human *ESRRA* SMART pool of three sequences: GGCCUUCGCUGAGGACUUA / GCGAGAGGAGUAUGUUCUA/GGGUGGGCAUGC UCAAGGA) and control sequences (ON-TARGET plus Human Non-targeting pool of three sequences: UGGUUUACAUGUCGACUAA/UGGUUUACAUGUU GUGUGA/UGGUUUACAUGUUUUCCUA) (Dharmacon) and mRNA were extracted at 72 h after transfection. Conditioned medium from PC3 and PC3c clones (used on primary calvaria cells culture) and PC3 parental cells treated with the XCT-790 at 10^−6^ M (Sigma) was obtained after 48 hours in α-MEM supplemented with 0.5% of serum, then filter sterilized and proteins quantified to use equal concentration of proteins for each conditions (25 μg). Dimethyl sulfoxide (DMSO) was used as control (CT). PC3-ERRα cells (pool of three clones) were treated with the inverse agonist XCT-790 at 10^−6^ M (Sigma) for 48 h. DMSO was used as control (CT).

### Osteoclastogenesis assay

Bone marrow cells from 6-week-old OF1 male mice were cultured for 7 days as previously described [22]. Pools of PC3-CT/PC3-ERRα clones or PC3c-CT/PC3c-ERRα or ACE-1-CT/ ERRα /AF2 were plated (10^4^ cells) on day 1 on bone marrow cells culture in presence of *rankl* and *m-csf*. Mature osteoclasts were stained for TRAP activity (Sigma-Aldrich). Multinucleated TRAP-positive cells containing three or more nuclei were counted as osteoclasts. Results were plotted as the mean ± SD of three wells for each condition and were representative of two independent experiments.

### Osteoblastogenesis assay

Cells were enzymatically isolated from the calvaria as described previously [31]. Mouse calvaria cells were continuously exposed (21 days) to conditioned medium isolated from cultures of PC3 pooled clones or PC3c clones. Wells were stained with von Kossa stain for ALP and bone nodules were counted on a grid [31]. Results were plotted as the mean number of nodules ± SD of three wells and were representative of two independent experiments. The murine osteoblastic MC3T3-E1 cell line was cultured for 2 weeks with pooled PC3-CT versus PC3-ERRα clones or ACE-1-CT/ERRα/AF2 that were plated (10^4^ cells) on day 1 for 2 weeks. mRNA were then extracted and osteoblastic markers expression addressed by real-time PCR.

### Immunohistochemistry

Tibia bearing metastases were fixed and embedded in paraffin. IHC analysis was performed by incubating sections overnight with rabbit monoclonal against human and mouse anti-*VEGF-A* (1/50) (Abcam), rabbit polyclonal antibodies against human and mouse *WNT5a* (1/70)(Abcam), goat anti-mouse *postn* (1/200)(R&D), mouse monoclonal anti- human/mouse *TGFb1* (1/40) (R&D), rabbit polyclonal anti-mouse vimentin(1/50)(Biovision) and mouse monoclonal anti-human/mouse *ERRα* antibody (1/50)(Santa Cruz) [22]. Sections were then incubated with HRP-conjugated anti-mouse (Dako), anti-rabbit (Dako) or the anti-goat (Jackson) antibodies for 1hour and stained using 3,3′-diaminobenzidine (Dako). Counterstaining was performed using Mayer's hematoxylin (Merck).

### Immunoblotting

Protein extraction and immunoblotting were performed as described previously [22]. As the mouse monoclonal anti-human/mouse *ERRα* antibody (Santa Cruz) did not work in Western-blots, we used a rabbit monoclonal antibody against *ERRα* (Epitomics) (1:400) [22] and a rabbit polyclonal antibody against *GAPDH* (Abcam) (1:10,000) (Amersham) as a protein loading control.

### Real time RT-PCR

Total RNA was extracted with Trizol reagent (Life Technologies) and 2 μg was reverse-transcribed using Superscript^™^II (Life Technologies) [22]. Real-time PCR was performed on a Mastercycler-ep-Realplex (Eppendorf) with primers specific to human and mouse genes (Supplementary Table S2) using Quantifast SYBR Green (Life Technologies). PCR was carried out according to the manufacturer's instructions. Melting curve analysis was used to verify that a single peak was obtained for each product with a 95–100% PCR efficiency (Roche software). Relative gene expression levels were normalized according to the Ct value of the housekeeping gene encoding the ribosomal protein L32 and results were expressed as fold differences equal to 2^−ΔΔCt^.

### Statistical analysis

Statistical analysis was performed using GraphPad Prism software (San Diego,USA). Pairwise comparisons were tested using a non-parametric Mann-Whitney *U* test for bone metastases. *In vitro* data were analyzed using ANOVA and Student's *t*-tests to assess the differences between groups. Results of *P* < 0.05 were considered significant.

## SUPPLEMENTARY MATERIALS FIGURE AND TABLES


