# Supplementary material for: Estrogen related receptor alpha in castration-resistant prostate cancer cells promotes tumor progression in bone
Source: Oncotarget. 2016 Oct 20;7(47):77071–86. doi: 10.18632/oncotarget.12787 (PMC5363569; doi:10.18632/oncotarget.12787)
Supplement: Supplementary file 1 [file oncotarget-07-77071-s001.pdf]

Estrogen related receptor alpha in castration-resistant prostate cancer cells promotes tumor progression in bone

Supplementary Materials

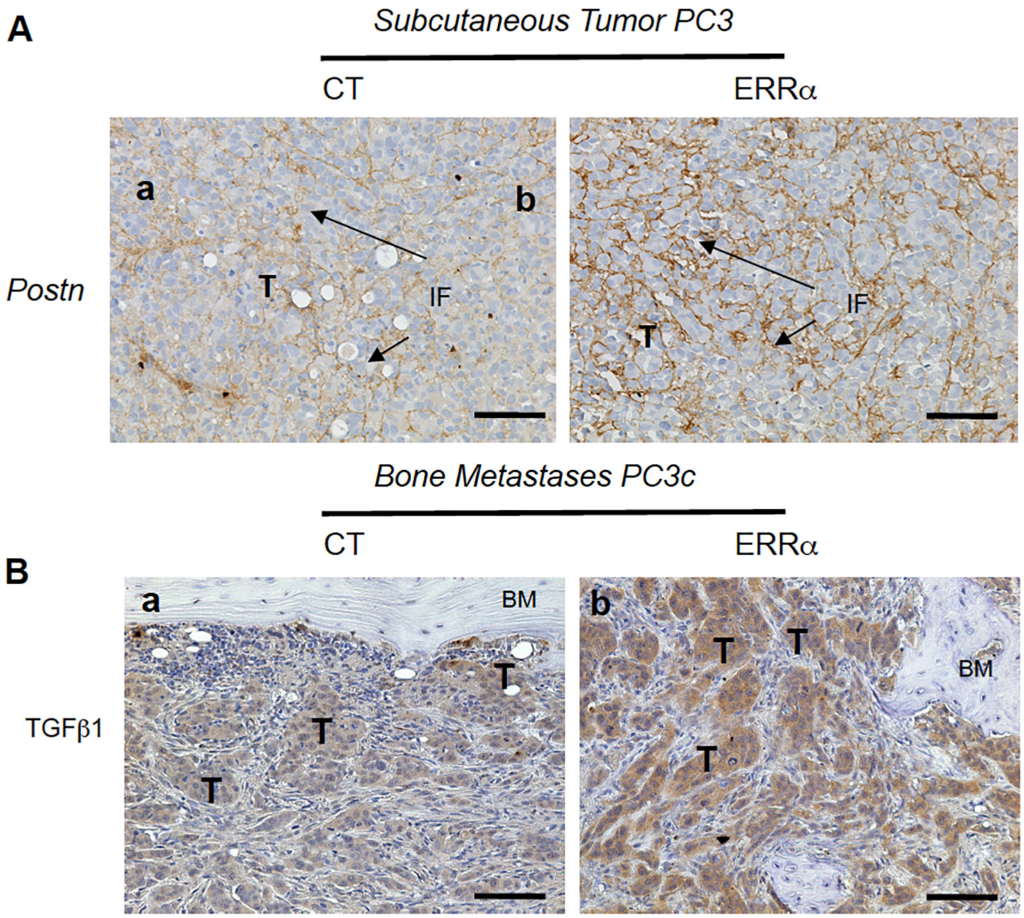

Supplementary Figure S1: (**A**) Immunohistochemistry showing the stimulation of *postn* in IF in SC tumor in PC3-ERRα (b) compared to PC3-CT (a). (**B**) Reinforcing the PC3 model, *TGFβ1* protein expression level was also up-regulated in tumor *in vivo* in bone lesions induced by PC3c-ERRα (ERRα)(b) compared PC3c-CT (CT) cells (a). Bar = 200 μm, T: Tumor; BM: Bone Matrix; IF: infiltrating fibroblasts.

Supplementary Table S1: General information on the public datasets used for meta-analysis on *ERRα* in CRPC (Figure 1A–1C) and to obtain correlation between *ERRα* and *VEGFA, WNT5A, POSTN* and *TGFβ1* expression levels in bone metastases (GSE29650, GSE41619 and GSE74685) and/or in primary CRPC tumor (GSE1034, GSE6919 and GSE2269)

			Number of samples (N)			
**Database**	**Description**	**Analysis Platform**	**Primary tumour**	**Adjacent/ stroma**	**Normal Prostate**	**Bone Metastases**
GSE21034	Human primary and metastatic prostate cancer samples and control normal adjacent benign prostate. CRPC samples are defined by biochemical recurrence and metastases.[24]	Affymetrix Human Exon 1.0 ST Array	150	29	N/A	N/A
GSE6919	Patients with androgen-insensitive prostate cancer and normal prostate tissue from organ donors and adjacent tissue.[25]	Affymetrix Human Genome U95C Array and Version 2 Array	65	63	18	N/A
GSE32269	Primary prostate cancer tissue from patients with bone metastatic and localised disease. PCa samples are defined as AR positive (PCa) or AR negative (CRPC) based on the presence of (bone) metastases. No bone metastases were collected, only primary tumour tissue available.[26]	Affymetrix Human Genome U133A Array	51	N/A	4	N/A
GSE29650	Bone metastases were obtained from CRPC patients.[34]	Illumina HumanHT-12 V3.0 expression beadchip	N/A	N/A	N/A	30
GSE41619	Osteoblastic and osteolytic bone metastases from patients with CRPC.[35]	Agilent-016162 PEDB Whole Human Genome Microarray 4x44K	N/A	N/A	N/A	14
GSE74685	Metastases collected from autopsies of patients who died from metastatic CRPC.[36]	Agilent-016162 PEDB Whole Human Genome Microarray 4x44K	N/A	N/A	N/A	20

Supplementary Table S2: SiRNA sequences and human and mouse primers and PCR using conditions

**ERRa-SiRNA**	GGCCUUCGCUGAGGACUUA – GCGAGAGGAGUAUGUUCUA - GGGUGGGCAUGCUCAAGGA
**Control**	UGGUUUACAUGUCGACUAA – UGGUUUACAUGUUGUGUGA - UGGUUUACAUGUUUUCCUA

**Gene Human**	**Primers (PCR)**	**PCR cycles**	**T (°C)**	**Size (bp)**	**Reference**
L32	CAAGGAGCTGGAAGTGCTGC<br>CAGCTCTTTCCACGATGGC	25	64	100	NM_000994.3
ERRa	GGAGGCCCCCGGAAGACAGC<br>TCACAGAGGGTAGCCACGGCT	40	65	134	NM_001282451.1
WNT5A	GCAGGACGGTGTACAACCTGGC<br>AGCCGCATGGCCGCCGCGCTG	40	62	153	NM_003392.4
VEGF-A	AGGAGGAGGGCAGAATCA<br>TCTATCTTTCTTTGGTCTGCATT	30	60	315	NM_001025366.2
TGFb1	AGAAGCGGTACCTGAACCCG<br>GCCGGTAGTGAACCCGTTG	30	57	303	NM_000660.5

**Mouse**	**Primers (PCR)**	**PCR cycles**	**T (°C)**	**Size (bp)**	**Reference**
L32	CAAGGAGCTGGAGGTGCTGC<br>CTGCTCTTTCTACAATGGC	30	59	100	NM_172086
rankl	GTGGTCTGCAGGATCGCTCTG<br>CGCTGGGCCACATCCAACC	40	63	286	NM_011613.3
opg	TGTGTGACAAATGTGCTCC<br>GTCTCACCTGAGAAGAACCC	35	59	340	NM_008764.3
trap	TTCCTCCAGGATGGATTCATGG<br>CTGAAGATACTGCAGGTTGTGG	40	61	760	NM_001102405.1
ck	TTAATTTGGGAGAAAAACCT<br>AGCCGCCTCCACAGCCATAAT	40	56	400	NM_007802.4
caII	GCTCATCTGATGGCCAGGG<br>TAGCGGCTGAGCTGGACGCC	40	59	489	NM_009801.4
rank	TGCTCCTCTTCATCTCTGTGGTAGTAGTGG<br>TGGAGTGAAGTCTGCGCCCCACCCTGCTCC	40	61	1995	NM_009399.3
alp	CCCGAATCCTTAAGGGCCAG<br>TATGCGATGTCCTTGCAGC	35	59	409	NM_007431.3
bsp	TGCCTACTTTTATCCTCCTCTG<br>ACCCGAGAGTGTGGAAAGTG	40	59	201	XM_011252013
ocn	TGACAAAGCCTTCATGTCCA<br>GAGAGGACAGGGAGGATCAA	40	59	276	NM_007541.3
postn	AATGCTGCCCTGGCTATATG<br>GTAGTGGCTCCCACAATGC	40	55	103	NM_015784.3
